# Male breast cancer: diagnosis stages, treatment and survival in a country with limited resources (Burkina Faso)

**DOI:** 10.1186/s12957-017-1297-y

**Published:** 2018-01-11

**Authors:** Nayi Zongo, Smaïla Ouédraogo, Nina Korsaga-Somé, Ollo Roland Somé, Naïma GO, Edgar Ouangré, Maurice Zida, Gilbert Bonkoungou, Aimé Sosthène Ouédraogo, Aboubacar Hirrum Bambara, Bambara Augustin Tozoula, Si Simon Traoré, Ahmadou Dem, Pascal Niamba, Adama Traoré, Adama Sanou, Danielé Grazziotin Soares, Jean-Pierre Lotz

**Affiliations:** 1 Division of General surgery, University Hospital of Ouagadougou, Ouagadougou, Burkina Faso; 2 Division of Dermatology and Venerology, University Hospital of Ouagadougou, Ouagadougou, Burkina Faso; 3 Division of Pathologic Anatomy, University Hospital of Ouagadougou, Ouagadougou, Burkina Faso; 4 Oncology Institute Joliot Curie of Dakar (Senegal), Dakar, Senegal; 5 Alliance for Research in Cancerology - APREC, Medical Oncology Service, Tenon Hospital, Paris, France; 6 Tenon Hospital, division of Onco-Hematology, University Hospitals of Eastern Paris, APHP, Pierre and Marie Curie University, Paris, France; 7 Division of Epidemiology and Public Health, Yalgado Ouédraogo University Hospital of Ouagadougou, Ouagadougou, Burkina Faso; 8 Department of General Surgery, Yalgado Ouédraogo University Hospital of Ouagadougou, Ouagadougou, BP 7021 Burkina Faso

**Keywords:** Cancer, Breast, Man, Surgery, Survival

## Abstract

**Background:**

Male breast cancer is a rare and less known disease. Therapeutic modalities affect survival. In Burkina Faso, male breast cancers are diagnosed in everyday practice, but the prognosis at short-, middle-, and long-term remains unknown. The objective of this study is to study the diagnosis stages, therapeutic modalities, and 5-year survival in male breast cancer at the General Surgery Unit of Yalgado Ouedraogo University Hospital from 1990 to 2009.

**Methods:**

A cohort longitudinal study concerning cases of breast cancer diagnosed in man. Survival was assessed using the Kaplan–Meier method and survival curves were compared through the LogRank test.

**Results:**

Fifty-one cases of male breast cancer were followed-up, i.e., 2.6% of all breast cancers. Stages III and IV represented 88% of cases. Eleven patients (21.6%) were at metastatic stage. Patients were operated in 60.8% of cases. The surgery included axillary dissection in 25 (80.6%) out of 31 cases. Lumpectomy was performed on 6.5% of patients (2 cases). Fifteen (29.4%) and 11 (21.6%) patients underwent chemotherapy and hormonal therapy, respectively. The FAC protocol was mostly used. Radiation therapy was possible in two cases. The median deadline for follow-up was 14.8 months. A local recurrence was noticed in 3.2% of cases. The overall 5-year survival rate was 49.9%. The median survival was over 5 years for stages I and II. It was 54 down to 36 months for stages III and IV.

**Conclusion:**

Diagnosis is late. The lack of immunohistochemistry makes it difficult to define the proportion of their hormonal dependence. Surgery is the basic treatment. Five-year survival is slow and the median survival depends on the diagnosis stage. It can be improved through awareness-raising campaigns and the conduct of individual screening.

## Background

Male breast cancer is a rare and less known disease [1, 2]. In western countries, it represents about 1% of all breast cancers and less than 1% of all male cancers [3, 4]. It is suspected to be mostly frequent in Sub-Saharan Africa, reaching 4 to 13% of all breast cancers [5]. Tumors with sizes T3–T4 represent 7% of cases in western countries [6], against 75.4 to 100% in Africa [7, 8].

Modeled on the treatment of female breast cancer, the treatment of male breast cancer is done according to the tumor size [2, 4]. In western countries, patients are treated with surgery, chemotherapy, hormonal therapy and radiation therapy [9, 10] while in Africa, surgery remains the only treatment in almost all cases [7, 8, 11].

Therapeutic modalities affect survival in male breast cancer. It is like that of female breast cancers at equal stage [6]. The 5-year survival varies between 43 and 85% in western countries [6, 12] against 7 and 63% in Africa [13, 14].

In Burkina Faso, male breast cancers are diagnosed in everyday practice, but the prognosis at short-, middle-, and long-term remains unknown. The absence of a radiation therapy center and the high cost of chemotherapy compared to incomes of the population make surgery the most affordable treatment. In this context, we are intending, through this work, to study the diagnosis stages, therapeutic modalities and the 5-year survival of male patients followed-up for breast cancer.

## Methods

### Type and period of study

This is a cohort longitudinal study concerning cases of male breast cancers diagnosed and followed-up between 1 January 1990 and 30 May 2015.

### Site of study

The study was conducted in Burkina Faso, a country with limited resources located at the heart of West Africa. The data collection was done at the General Surgery Unit of Yalgado Ouedraogo University Hospital of Ouagadougou (CHU-YO). CHU-YO is the highest level for health evacuations in Burkina Faso. The General Surgery Unit represents with its Cancer Section, the National reference unit that handles male cancers.

### Population of study

For this work, we have considered all cases of male breast cancers diagnosed that have their therapeutic schema and follow-up data available. Clinical files not containing enough information were excluded (3 cases).

### Procedures for data collection

Clinical files of patients, admission files and operating reports were the sources of the data. For each patient, we collected data that enabled defining the stages of the cancer, such as the tumor size, the presence of satellite lymph nodes (axillary, sub-clavicular, supra-clavicular), and metastasis. The fixed or non-fixed aspect of the tumor and/or satellite lymph nodes was assessed.

The therapeutic modalities (surgery, chemotherapy, radiation therapy, hormonal therapy) were noted. We also had interest in the duration of the follow-up, survival without recurrence, and the overall survival of the patients.

### Data management and strategy of analysis

The data were typed and analyzed with the software SPSS 2000. We proceeded with the staging of cancers using the WHO 2009 TNM (tumor-nodes-metastasis) classification. It considers the tumor size and the presence of satellite lymph nodes and metastasis. The remote search for metastasis was performed by a thoraco-abdominal-pelvic scanner or optionally by associating chest X-ray and abdominal-pelvic CT. Tumors were classified from stages I to IV. The prognosis factors of male breast cancers, such as age and tumor size were considered. Survival was calculated according to the Kaplan–Meier method and the comparison of survival curves was made possible thanks to the LogRank test. The significance threshold was set at 0.05.

### Ethical aspects

The study was authorized by the Management and the General Surgery Unit of Yalgado Ouedraogo University Hospital. The data collection was done anonymously and confidentiality was respected for all the patients.

## Results

### General data of the population of study

From 1990 to 2009, 51 cases of male breast cancer were followed-up in the General Surgery Unit of Yalgado Ouedraogo University Hospital. With an annual range of 2.5 cases, they represent 2.6% of all breast cancers diagnosed within the same period (1988 cases).

The patients had a median age of 60.9 years ± 8.4. The age group between 61 and 70 years was the most represented with 33% of cases (Table 1). Histories of breast cancer and ovarian cancer were noted in the mother in 3 and 1 cases, respectively. No genetic studies could be conducted. Three patients were having diabetes.

**Table 1:** Clinico-pathological features

	Category	Number	Proportion %
Age (years)	< 60	16	31.4
	60–70	18	35.3
	> 70	17	33.3
Primary tumor	T1	05	9.8
	T2	07	13.7
	T3	12	23.5
	T4	27	53
Lymphnode	N0	14	27.4
	N1	30	58.8
	N2	7	13.7
Stage	I	05	9.8
	II	06	11.7
	III	29	56.9
	IV	11	21.6
Lymphnode status	pN0	2/25	–
	pN+	23/25	–
Hormone receptors	ER+	12/14	–
	PR+	12/14	–

*ER* estrogen receptor, *PR* progesterone receptor

The tumors were epithelial (ductal and lobular carcinoma) in 91.1% of cases. Rare tumors like fibrosarcoma, mammary lymphoma, and Darier–Ferrand dermatofribrosarcoma of the breast were each noted in one case. For the Scarff Bloom Richardson histological prognosis grading, we noted 6 patients ranked SBRI, 33 patients ranked SBRII, and 12 patients ranked SBRIII. Immunohistochemistry was obtained for 14 patients. Hormonal receptors of estrogen and progesterone were positive for 12 patients and treble negative for the two others (RO-, RP-, and HER2-).

### Diagnosis stages (cTNM)

**Fig. 1:**
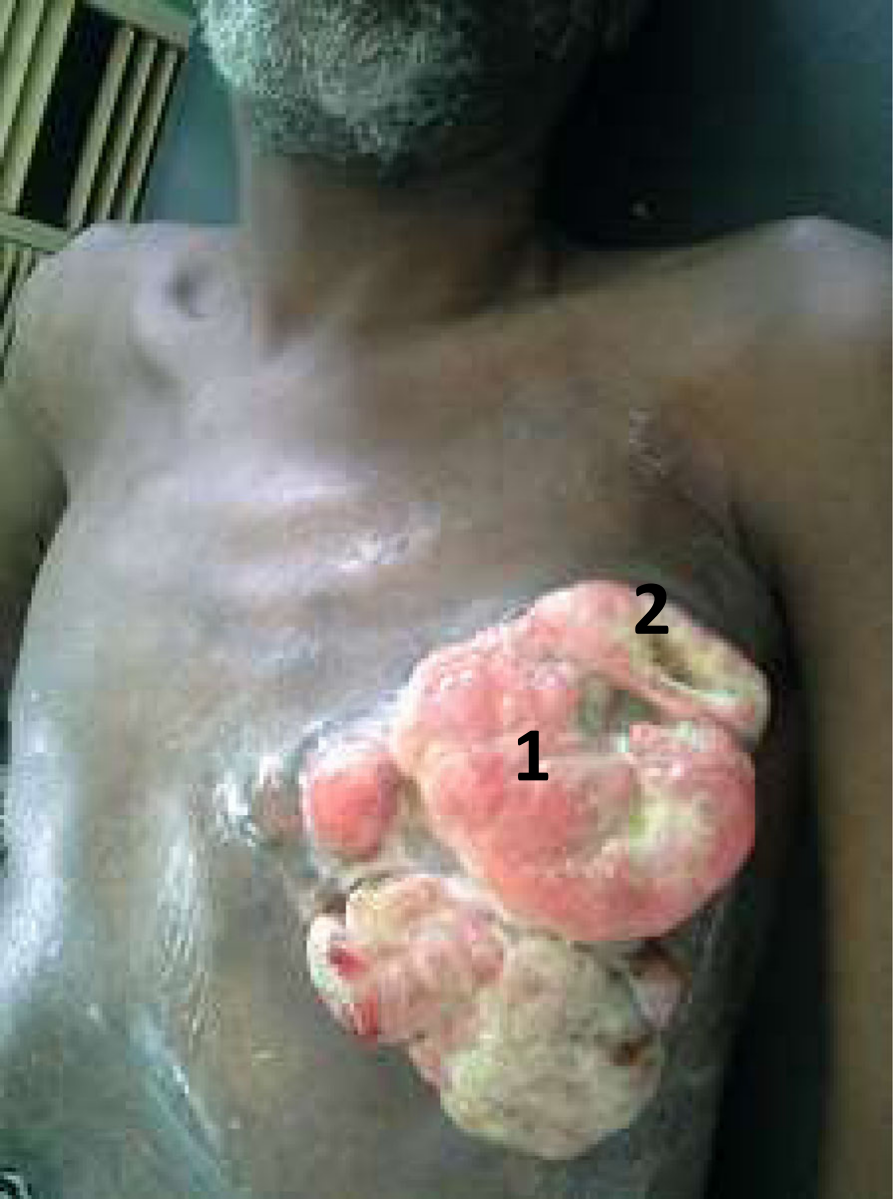
Ulcero-bourgeoning and necrosic tumor treated by septic mastectomy. 1: ulcero-granulating tumor; 2: Necrotic zone

**Fig. 2:**
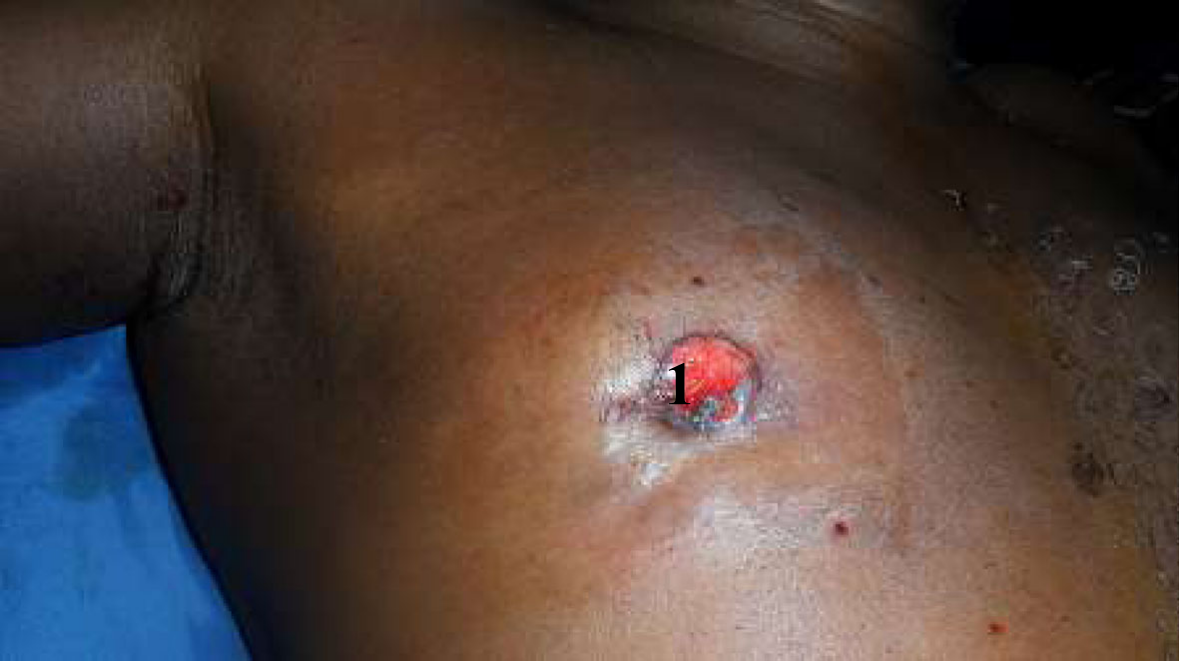
Paget’s disease of the right nipple. 1: Paget’s disease of the right nipple

The tumor sizes varied between 2 and 20 cm. T3 and T4 represented 76.5% of cases (Table 1). For the T4, it was mostly ulcerated tumors not fixed to the chest wall in 16 cases over 27. Two patients had Paget’s disease of the nipple (Figs. 1 and 2).

Lymphadenopathies were noticed at the homolateral axillary level in 37 cases (Table 1). None of the patients had supra-clavicular lymphadenopathies (N3c).

Among the patients without palpable lymphadenopathies (N0), we had low lymphophilic tumors like Darier–Ferrand dermatofibrosarcoma (1 case) and fibrosarcoma (1 case).

Stages I and II represented 21.5% of cases (Table 1). Eleven patients had secondary localizations. There were chest metastasis in six cases and hepatic metastasis in three cases. One patient had bone metastasis and another one chest, hepatic, and bone metastases.

#### Therapeutic aspects

The patients were operated in 31 cases. Twenty-seven patients were operated to be cured. In four cases (12.9%), septic mastectomy was performed. The surgery included axillary dissection in 25 cases (80.6%). The surgery of non-lymphophilic tumors (fibrosarcoma and dermatofibrosarcoma) was limited to mastectomy (Table 2). Surgical gestures in our series were stratified into two periods.

**Table 2:** Surgical procedures for male breast cancer *n* = 31

Surgical procedures	Number	Proportion %
Mastectomy with axillary dissection	25	80.6
Mastectomy alone	4	12.9
Lumpectomy	2	6.5
Total	31	100

From 1990 to 2000, of 20 male patients with breast cancer, 14 were operated. A radical Patey’s mastectomy with axillary lymph node dissection was performed in four cases. Seven patients underwent Madden’s mastectomy (modified Patey). Mastectomy without dissection was performed for breast fibrosarcoma and two septic mastectomies for necrotic breast cancers. From 2000 to 2009, 17 over 31 male patients with breast cancers were operated. Mastectomy with dissection of lymph nodes according to Madden (modified Patey) was performed in twelve (12) cases (12/17), septic mastectomy in two (2) cases, and simple mastectomy in one (1) case of Darier–Ferrand dermatofibrosarcoma of the breast. Lumpectomy was performed in two (2) cases of mammary carcinoma (Table 2).

There was an indication of chemotherapy and radiation therapy in 48/51 cases, hormonal therapy for 12/14 patients whom we could get the immunohistochemistry.

Of the 20 patients diagnosed between 1990 and 2000, three (3) patients could afford chemotherapy. It was adjuvant for one (1) patient using the FEC protocol (5-fluoro-uracil, epirubicin, cyclophosphamide) and for a palliative purpose for the two (2) other metastatic patients, based on the CMF protocol (cyclophosphamide, methotrexate, 5-fluoro-uracil). Hormonal therapy with tamoxifen was used in one (1) case.

From 2000 to 2009, the FAC protocol (5-fluoro-uracil, adriamycin, cyclophosphamide) was used on the 12 patients that received chemotherapy. Docetaxel was used in addition to the FAC protocol on six (6) patients. Chemotherapy was neo-adjuvant in five (5) cases. It was neo-adjuvant and adjuvant in four (4) cases, adjuvant in one (1) case and palliative in two (2) cases. The median number of cures was 2 with extremes from one (1) to eight (8) cures. Tamoxifen was used in eleven (11) cases. Two patients underwent radiation therapy outside the country.

### Evolutionary aspects

The median deadline of follow-up was 40.8 months, with extremes from 1 to 60 months. We noted a local recurrence rate at 3.2% (2/31 operated). The overall 5-year survival was at 49%. Five-year mortality was 5/16, 7/18, and 9/17 for those under 60, 61 to 70 years, and the over 70, respectively.

**Fig. 3:**
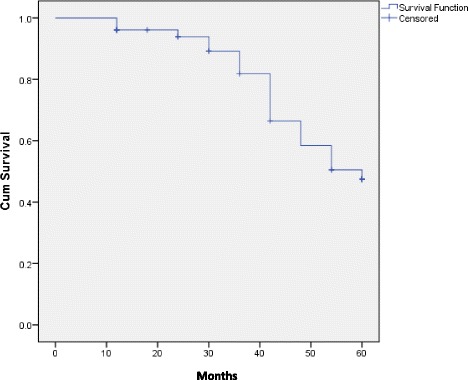
Overall survival in the cohort of 51 patients with breast cancer diagnosed between 1900 and 2009 in Burkina Faso

**Fig. 4:**
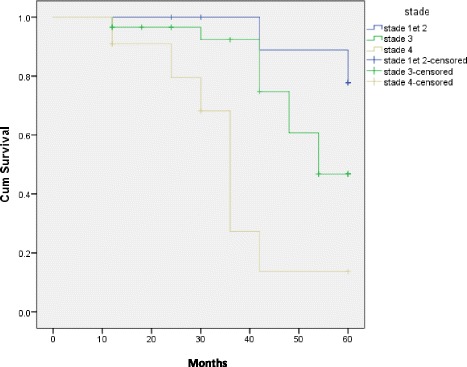
Survival per tumor stage. Censored patients are indicated on the curve

The median survival was superior to 5 years for stages I and II. It was between 54 and 36 months for stages III and IV, respectively. The LogRank (Mantel-cox) test on survival according to diagnosis stages revealed some statistically meaningful difference (chi-squared = 16.3, *p* < 0.0001). Figures 3 and 4 represent the overall survival and the survival by stage with a decline of 5 years.

Five-year mortality was 5/15 and 20/36, respectively, for patients who got chemotherapy and those who did not.

By specifying (operated, non-operated), the overall survival was at 70% for patients that were operated and 40% for the non-operated RD f(*p* = 0.001).

## Discussion

Systematic screening, awareness-raising, and the strong involvement of women has enabled to improve the diagnosis deadlines of female breast cancers [15]. On the contrary, the scarcity of breast cancers and the absence of similar motivation of males still result in long deadlines of diagnosis [15, 16]. Nevertheless, the extent of this late diagnosis is related to the level of development or organization of countries. Thus, there is a contrast between the tumor size in our series and those of western countries [2, 6, 10]. In fact, in western countries, diagnosis is made at early clinical stages in 80% of cases. Tumors with a big size (T3–T4) represent only 7 to 13% of cases [6, 10]. However, though it is a superficial and quite small organ in man, and can easily be examined during clinical examinations, diagnosis is late in African series [7, 8, 11, 13]. Tumors with sizes T3–T4 represent up to 75.4 to 100% of cases [7, 8]. The big size of these tumors may explain the frequent invasion of lymph nodes in male breast cancer [17, 18]. It is an evidence of a locoregional progression of the tumor and necessarily requires axillary dissection. In our series, 72.5% of the patients had lymphadenopathies that were palpable during the diagnosis. In the African literature, there were lymphadenopathies in 61.5 to 88.9% of cases [19].

Eleven (21.6%) of our patients had secondary localizations at the time of diagnosis. In African series, the presence of metastasis noticed during diagnosis is common. The proportion of metastatic forms varies between 15 and 26.3% of cases [7, 8]. In contrast, it is low or even null in European countries (0–2%) [10, 20].

In our series, diagnosis is late with stage III in 56.9% of cases. In the African literature, stages III and IV are the most represented [7, 11, 13], i.e., 61.4 to 93.0% of cases. In the USA, at the time of diagnosis, only 12.8 to 15.6% of patients are at stage III and 5.4 to 8.9% at stage IV [12, 21]. In Japan, all the eight cases published by Horimoto were at a stage inferior to III [22].

Late diagnosis in our context (Burkina Faso) is said to be mostly due to ignorance and poverty. Patients first go and see traditional healers who are more affordable and the hospital is the last resort, mostly when they face some complications [8]. This may explain the frequency of stage IV (21.6%) and even necrotized and foul-smelling tumors (1.9%) which required septic surgery in our series. The absence of enough cancer experts and institutes specialized in cancer treatment could also contribute to exacerbate this late diagnosis. The holding of awareness-raising campaigns and/or screening within the male population could allow for early diagnosis. This will significantly reduce the number of lymphadenopathies, a sign of a regional extension at the time of diagnosis. Except this, individual screening can be done.

Apart from this late diagnosis, male breast cancer raises an issue of therapeutic indication. In fact, because of the small size of samples, a specific treatment of male breast cancers does not seem to emerge. It is therefore modeled on that of women [2, 4]. In rich countries which are better organized and equipped, patients enjoy a well codified set of treatments. For example, in the USA, patients of Akkamma’s series were treated with surgery (95%), chemotherapy (54.5%), hormonal therapy (61%), and radiation therapy (34%) [9]. In France, in Oger’s series, patients were also treated by surgery (98%), chemotherapy (37%), hormonal therapy (92%), and radiation therapy (75%) [10].

In Africa, surgery remains practically the only treatment in almost all the series published [7, 8, 11].

Traditionally radical mastectomy (HALSTED) was the reference treatment in male breast cancer due to the frequent invasion of the pectoral major muscle. Increasingly, it leaves space for modified radical mastectomy (Patey and modified Patey) that gives the same results with less postoperative complications [23–25]. Radical mastectomy (HALSTED) and modified radical mastectomy (PATEY) represent together, more than 70% of surgical procedures [16]. Nowadays, modified radical mastectomy is the standard treatment [23, 18, 26].

However, the surgery of male breast cancer has become less and less invasive. The most recent studies mention lumpectomy and sentinel lymph node technique which give the same therapeutic results as radical surgery for tumors with sizes T1 and T2 [9, 18, 27, 28]. The sentinel lymph node technique would enable like in women, reduce morbidity related to axillary dissection for small tumors of less than 2.5 cm [7, 17]. It must be noted in our context that modified radical mastectomy and lymph node dissection (80.6%) remain the first resort against male breast cancer, due to late diagnosis. Breast conserving surgery was performed in only two (2) cases of our series. This is due to the big sizes of the tumors mostly requiring radical surgery and further treatment.

The high hormonal dependence (75–93%) in male breast cancer makes hormonal therapy a key element of the treatment [29]. Tamoxifen is the choice molecule compared to anti-aromatases. It is the standard adjuvant treatment and is believed to improve overall survival [13, 16]. However, it is less invasive than chemotherapy, on these already fragile areas (median age superior to 60 years [6]. Taxanes are used in case of invasion of lymph nodes [16]. In our series, the low use of hormonal therapy (11/51 cases) can be explained by the impossibility to carry out immunohistochemistry examination locally. Apart from systemic treatments (hormonal therapy, chemotherapy), the locally advanced nature of the tumors demands radiation therapy.

It consists in administering 50 Gy in 25 fractions [13, 16]. Its indication remains a large tumor of more than one (1) cm, invasion of lymph nodes, centrally localized tumor (areolar), muscle damage, and conserving surgery associated with a high risk of unacceptable local recurrence [16]. The low amount of tissue in the mammary gland of man makes that space limited and can be an indication for radiation therapy [30]. The absence of a radiation therapy center in Burkina Faso and the high cost of evacuations to countries where there are renders this therapeutic modality unaffordable for our patients. Yet, it improves survival without local recurrence, even though it does not seem to have an impact on the overall survival [31–33].

Despites the absence of radiation therapy in our work environment, only two cases (2/31 cases) of local recurrence were noted. This could be due to the radical nature of our surgical approach that permits resections R0.

This best survival without local recurrence contrasts to the weakness of the overall survival. In fact, the 5-year survival in our series was at 49%. It varies between 7 and 92% in the literature [16]. In western countries, it is at 42 to 85% and is almost always superior to 70%, according to recent publications [16]. In Asia, it varies between 27 and 92%. In Africa, it is at 7 to 63% [13–15]. The major survival factors reported in the literature are mostly the tumor size, the damage of lymph nodes, and age [6]. Our data were consistent. Thus, survival depended on the diagnosis stage. The more the stage is advanced, the less the survival is good (chi-squared = 16.3, *p* < 0.0001). The literature reports 5-year survival at 75 to 100% for patients at stage I, 50–80% for stage II, and 30–60% for stage III [19, 28, 34, 35]. Age is described as an independent survival factor [6, 36]. The 5-year survival is at 87, 69, and 24% for patients below 50, from 50 to 70, and over 70, respectively [6]. In our series, the more the age is advanced, the higher the mortality is, with a proportion of 1.3% between the over 70 and the under 60 years.

## Conclusion

The diagnosis of male breast cancer remains late at Yalgado Ouedraogo University Hospital of Ouagadougou. The absence of immunohistochemistry does not enable defining the proportion of their hormonal dependence. Surgery is the basic treatment. Chemotherapy, though increasingly used, remains limited in the choice of molecules and the number of cures, due to the cost of cytotoxics. Radiation therapy is unaffordable because it is not available in Burkina Faso. The 5-year survival is slow and the median survival still depends on the diagnosis stage. Awareness-raising campaigns and the organizing of individual screening could enable reduce lateness in diagnosis and improve patients’ chances of recovery.

## Abbreviations

CMF: Cyclophosphamide, methotrexate, 5-fluoro-uracil
cTNM: Clinical tumor-nodes-metastasis
FAC: 5-fluoro-uracil, adriamycin, cyclophosphamide
FEC: 5-fluoro-uracil, epirubicin, cyclophosphamide
FIGO: International federation of gynecology and obstetrics
HER2: Herceptin receptor
RO, RP: Oestrogen receptor, progesteron receptor
SBR: Scarf Bloom Richardson
TNM: Tumor nodes metastasis
